# Diet-Induced Metabolic Syndrome Reduced Heart Rate Variability and Increased Irregularity and Complexity of Short-Term RR Time Series in Rabbits

**DOI:** 10.3390/ani9080572

**Published:** 2019-08-18

**Authors:** Wilson M. Lozano, Conrado J. Calvo, Oscar J. Arias-Mutis, Ana Díaz, Luis Such-Miquel, Jichao Zhao, Antonio Alberola, Francisco J. Chorro, Manuel Zarzoso

**Affiliations:** 1 Department of Physiology, Universitat de València, 46010 Valencia, Spain; 2 Centro de Investigación Biomédica en red (CIBERCV), Instituto de Salud Carlos III, 28029 Madrid, Spain; 3 Unidad Central de Investigación de Medicina (UCIM), Universitat de València, 46010 Valencia, Spain; 4 Department of Physiotherapy, Universitat de València, 46010 Valencia, Spain; 5 Auckland Bioengineering Institute, The Univeristy of Auckland, 1010 Auckland, New Zealand; 6 Department of Cardiology, Hospital Clínico Universitario, 46010 Valencia, Spain

**Keywords:** metabolic syndrome, heart rate variability, arrhythmias, rabbit

## Abstract

**Simple Summary:**

In recent years, obesity and metabolic syndrome (MetS) have become more prevalent, owing to increased unhealthy habits and sedentary lifestyles becoming public health problems. Both conditions are linked with a higher prevalence of sudden cardiac death (SCD), but the exact mechanisms are not known. An autonomic nervous system imbalance can produce atrial and ventricular arrhythmias, which cause SCD, and this can be quantified by analyzing heart rate variability (HRV). We investigated HRV using time-domain, frequency-domain and nonlinear analyses during the development of MetS in rabbits and found HRV modifications that could be associated with the higher prevalence of SCD in this pathological condition.

**Abstract:**

Metabolic syndrome (MetS) has been linked to a higher prevalence of sudden cardiac death (SCD), but the mechanisms are not well understood. One possible underlying mechanism may be an abnormal modulation of autonomic activity, which can be quantified by analyzing heart rate variability (HRV). Our aim was to investigate the modifications of short-term HRV in an experimental rabbit model during the time-course of MetS development. NZW rabbits were randomly assigned to a control (*n* = 10) or a MetS group (*n* = 13), fed 28 weeks with control or high-fat, high-sucrose diets. After anesthesia, a 15-min ECG recording was acquired before diet administration and at weeks 14 and 28. We analyzed short RR time series using time-domain, frequency-domain and nonlinear analyses. A mixed-model factorial ANOVA was used for statistical analysis. Time-domain analysis showed a 52.4% decrease in the standard deviation of heart rate in animals from the MetS group at week 28, but no changes in the rest of parameters. In the frequency domain, we found a 9.7% decrease in the very low frequency and a 380.0% increase of the low frequency bands in MetS animals at week 28, whereas high frequency remained unchanged. Nonlinear analyses showed increased complexity and irregularity of the RR time series in MetS animals.

## 1. Introduction

Obesity and metabolic syndrome (MetS) are becoming a global epidemic worldwide, and their prevalence has dramatically increased during the last 15 years in both children and adults [1]. MetS is composed of a variable cluster of cardiometabolic risk factors and comorbidities, such as abdominal obesity, reduced HDL and elevated LDL cholesterol, elevated triglycerides, glucose intolerance, and hypertension, that portend high risk of both cardiovascular disease and type 2 diabetes. It is responsible for huge socio-economic costs, resulting morbidity and mortality in most countries [1,2]. Indeed, the different components of MetS have been linked, individually or combined, with a higher prevalence of sudden cardiac death (SCD) [3], but the exact underlying mechanisms remain poorly understood.

Heart rate responds dynamically to physiologic and pathologic perturbations. This cyclic fluctuation of RR intervals can be analyzed using heart rate variability (HRV), which can be used as a quantitative marker of autonomic activity [4]. One of the proposed mechanisms of cardiac arrhythmia leading to SCD is an abnormal modulation of autonomic activity. Indeed, experimental and clinical evidence has found an association between propensity for lethal arrhythmias and signs of either increased sympathetic/parasympathetic activity or reduced vagal activity [5,6,7]. 

Recently, we developed a diet-induced experimental model of MetS in New Zealand White (NZW) rabbits, which reproduced the main components of human MetS, namely, central obesity, a state of prediabetes characterized by impaired fasting glucose and glucose intolerance, mild hypertension, and alterations in the lipid profile (increased triglycerides and LDL, decreased HDL and no changes in total cholesterol) [8]. Our aim was to investigate the time-course modifications of short-term HRV in an experimental rabbit model of diet-induced MetS during its development in order to identify the early changes in HRV and its potential relationship with cardiac arrhythmias and SCD.

## 2. Material and Methods

### 2.1. Animals and Diets

Animal care and the experimental protocols used in this study complied with EU directive 2010/63 on the protection of animals used for scientific purposes and were approved by the Institutional Animal Care and Use Committee of the Universitat de València and the Ministry of Agriculture (2015/VSC/PEA/00049). Adult male NZW rabbits (*n* = 23) weighing 4.53 (0.21) kg and 16–18 weeks old at the beginning of the experimental protocol were used in the present study. The rabbits were housed in a room with humidity- (50 ± 5%) and temperature- (20 ± 1.5 °C) controlled conditions with a 12-h light cycle. MetS was induced as previously described [8,9]. Briefly, after an acclimation of 3 weeks in which rabbits were fed 120 g of standard rabbit chow (V2333-000, Ssniff, Soest, Germany), the animals were randomly assigned to a control (*n* = 10) or MetS group (*n* = 13). The control animals followed the same dietary regime, which has been shown to be appropriate for the maintenance of the adult rabbit [10]. Control diet contained 23.4% protein, 11.1% fat and 65.5% carbohydrates (2.7 kcal·g^−1^). Animals in the MetS group were fed ad libitum during 28 weeks with added high-fat (10% hydrogenated coconut oil, 5% lard; S9052-E020, Ssniff, Soest, Germany) and high-sucrose (15% dissolved in water) diet. High-fat chow was composed mainly by 15.7% protein, 43.1% fat and 41.2% carbohydrates (3.7 kcal·g^−1^), and the animals consumed 0.6 kcal·mL^−1^ in the drinking solution [8].

### 2.2. Electrocardiographic Study

We performed the electrocardiographic study at three different time points throughout the period of MetS induction: before high-fat and high-sucrose diet administration, at week 14, and at week 28. Animals were sedated (propofol 7 mg·kg^−1^ for induction and 2% isoflurane for maintenance) and, after a period of ten minutes of stabilization, 15-min electrocardiograms were obtained with an electrophysiology data acquisition system (Axoscope, Molecular Devices, Sunnyvale, CA, USA), configured as lead 1, with a sampling rate of 1 kHz.

### 2.3. Measurements and Calculations

Standard HRV parameters were quantified in the 15 min ECG recordings using a custom-made software, as previously described [4] and Kubios^®^ [11,12]. Briefly, after band-pass filtering between 0.5–250 Hz, baseline wander was removed using a bidirectional filtering strategy. Finite differential methods and wavelet transform were used for fiducial point estimation. R-peak detection was robustly estimated by parabolic fitting of the coiflet wavelet transform and detection of the maximum magnitude point. All R detections, defined as the peak of the ventricular activation wave, were supervised. RR series underwent high-pass filtering to ensure stationary results. Then, we quantified (1) Time-domain parameters: NN, SDNN, HR, SDHR, RMSSD, NN50, triangular index, and TINN (Appendix A Table A1); (2) Frequency-domain parameters: very low frequency (VLF), low frequency (LF), high frequency (HF) and LF-HF ratio and total power (Appendix A Table A2), using FFT (Welch periodogram window width 256 s and 50% overlap, interpolation rate 4 Hz). All these parameters were transformed using the natural logarithm (ln); and (3) non-linear parameters: Poincaré plot (SD1, SD2), detrended fluctuations (DFAα-1 and α-2), approximate entropy (ApEn), sample entropy (SampEn), multiscale entropy (MSE) and recurrence plot (DET, REC) analyses (Appendix A Table A3). The complexity index (CI_1–20_) was also determined as the mean of entropies on all 20 scales of MSE [4]. HRV values were expressed as the natural logarithm (Ln) to achieve a more normal distribution when necessary [13]. All the measurements were performed according to the standards determined by the Task Force of the European Society of Cardiology and the North American Society of Pacing and Electrophysiology [5].

### 2.4. Statistical Analysis

The values are reported as mean (SD) unless stated otherwise. Normality of data distribution was assessed using a Shapiro–Wilk test. A factorial ANOVA with one within-subjects factor (time: pre-diet, week 14 and week 28) and one between-subjects factor (group: control and MetS) was used for statistical analysis (SPSS version 24.0). When normality of data distribution was not met, Kruskal-Wallis and Friedman’s test were utilized. Differences were considered significant when *p* < 0.05.

## 3. Results

### 3.1. Time-Domain Analysis of Short-Term HRV

The time-domain analysis of short term (10–15 min) HRV showed a decreased SDHR in MetS animals at week 28 (*p* = 0.025; *r* = 0.490; Table 1). Regarding within-group comparisons, we found a decrease of this parameter in MetS animals at week 28 (*p* = 0.029; *r* = 348; Table 1), but no difference in controls. No differences were found when comparisons were made between two experimental groups in the rest of the parameters studied: NN, HR, SDNN, RMSSD, triangular index, NN50 and TINN (Table 1).

### 3.2. Modifications of Short-Term HRV in the Frequency Domain

**Figure 1:**
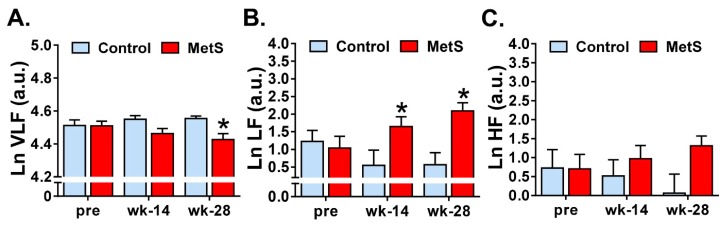
Frequency-domain parameters of short-term HRV analysis. Comparisons of LnVLF are shown in panel (**A**). Panel (**B**) shows the quantification in the low frequency (LF) band, whereas ln high frequency (HF) is depicted in panel (**C**) (log forms). Control *n* = 9, MetS *n* = 13. * *p* < 0.05 vs. control. Error bars: SEM.

Regarding frequency-domain analysis using FFT, differences between groups were observed in LnVLF (*p* = 0.020; η^2^_p_ = 0.254) and LnLF (*p* = 0.027; η^2^_p_ = 0.233). Indeed, when pairwise comparisons were analyzed, we did find a decrease in LnVLF in MetS animals at week 28 (4.6 (0.1) vs. 4.4 (0.1); *p* = 0.016; *r* = 0.521) (Figure 1A). On the other hand, LnLF increased in MetS group when comparisons were made at weeks 14 (0.5 (1.2) vs. 1.6 (1.0); *p* = 0.037; *r* = 0.457) and 28 (0.6 (1.0) vs. 2.1 (0.8); *p* = 0.002; *r* = 0.644) (Figure 1B). Within-group comparisons showed an increase in only LnLF at week 28 in MetS animals (0.98 (0.54) vs. 2.09 (0.83); *p* = 0.013; *r* = 0.399), but no difference was found in the control group.

No changes were found in the rest of the frequency-domain parameters studied: LnHF, normalized LF and HF powers, LF/HF balance and Ln of total power (Figure 1C, Table 2).

### 3.3. Nonlinear Analysis of Short-Term HRV

**Figure 2:**
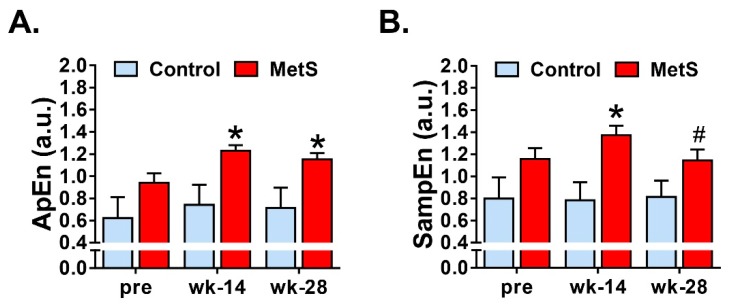
Nonlinear analysis of short-term HRV. Panel (**A**) shows changes in approximate entropy (ApEn). Sample entropy (SampEn) is displayed in panel (**B**) Control *n* = 9, MetS *n* = 13. * *p* < 0.05, ^#^ *p* = 0.07 vs. control. Error bars: SEM.

We then analyzed the complexity and irregularity of the RR time series. We did find differences between groups in ApEn (*p* = 0.002; η^2^_p_ = 0.399) and SampEn (*p* = 0.005; η^2^_p_ = 0.333). No difference was found in the control and MetS groups when comparisons within-groups were performed. Pairwise comparisons showed an increased ApEn in MetS animals at both weeks 14 (0.74 (0.54) vs. 1.23 (0.18); *p* = 0.007; *r* = 0.559) and 28 (0.71 (0.54) vs. 1.15 (0.2); *p* = 0.015; *r* = 0.514) (Figure 2A). Regarding SampEn, this parameter also increased in MetS group at week 14 (0.78 (0.48) vs. 1.37 (0.31); *p* = 0.002; *r* = 0.615) and tended to increase at week 28 (0.81 (0.44) vs. 1.14 (0.37); *p* = 0.072) (Figure 2B).

**Figure 3:**
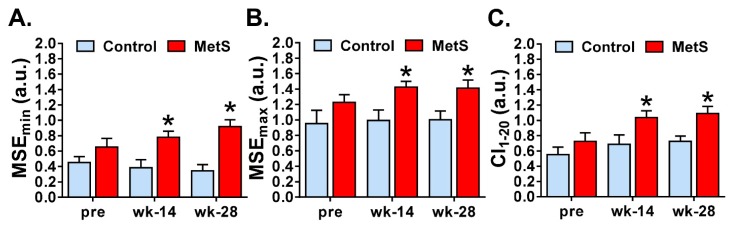
Multiscale entropy analysis of short-term HRV. Quantification of the minimum and maximum multiscale entropy (MSE_min_ and MSE_max_) is shown in panels (**A**) and (**B**) respectively. The quantification of complexity index (CI_1–20_) is depicted in panel (**C**) Control *n* = 9, MetS *n* = 13. * *p* < 0.05 vs control. Error bars: SEM.

With respect to the complexity of the short-term RR series determined with the multi-scale entropy, we found a significant effect of the factor “group” in MSE_min_ (*p* = 0.000; η^2^_p_ = 0.479), MSE_max_ (*p* = 0.007; η^2^_p_ = 0.312) and Complexity Index (CI_1–20_) (*p* = 0.012; η^2^_p_ = 0.275). When we looked into the pairwise comparisons in MSE_min_, we found an increase at weeks 14 (0.38 (0.31) vs. 0.78 (0.28); *p* = 0.006; *r* = 0.403) and 28 (0.34 (0.24) vs. 0.91 (0.32); *p* < 0.001; *r* = 0.731) (Figure 3A). The same trend was found in MSE_max_, which increased at both week 14 (0.99 (0.40) vs. 1.42 (0.27); *p* = 0.007; *r* = 0.555) and 28 (1.00 (0.34) vs. 1.41 (0.38); *p* = 0.017; *r* = 0.505) in MetS animals (Figure 3B). Finally, as expected, CI_1–20_ also increased in the experimental group at week 14 (0.68 (0.37) vs. 1.04 (0.32); *p* = 0.029; *r* = 0.467) and 28 [0.73 (0.20) vs. 1.10 (0.33); *p* = 0.009; *r* = 0.546] (Figure 3C). When looking at within-group comparisons, a significant increase was observed in MetS animals at week 28 in MSE_min_ (0.65 (0.41) vs. 0.92 (0.32); *p* = 0.043; *r* = 0.324), and at weeks 14 (0.72 (0.40) vs. 1.04 (0.32); *p* = 0.023; *r* = 0.363) and 28 (0.72 (0.40) vs. 1.09 (0.33); *p* = 0.001; *r* = 0.501) in CI_1–20_ when compared with pre-diet administration. No difference was found within the control group in any of the aforementioned parameters.

Poincaré analysis and SD1-SD2 quantification were performed to identify dynamic changes in heart rate, but this analysis showed no differences between groups (Table 3). In addition, we did not find any change in the rest of the nonlinear parameters used to quantify detrended fluctuations (DFA-α1 and DFA-α2) and the recurrent plot analysis (DET, REC), as shown in Table 3.

## 4. Discussion

This study includes a comprehensive analysis of HRV alterations, which is a non-invasive measure of cardiac autonomic function, found in a diet-induced model of MetS using short-term ECG recordings. The analysis of HRV is a very useful and inexpensive tool that enables the analysis of heart rate dynamics and the detection of any early stage indication of cardiac dysfunction. In our study, we used short-term (15-min) recordings to analyze HRV during the course of MetS development using high-fat, high-sucrose diet in NZW rabbits. The advantages of short term HRV analysis include easier usability and analysis, the ability to collect data in a very controlled environment, and better suitability for spectral analysis that require stationary conditions [5,14]. In our study, using conventional HRV analyses, we found significant alterations in heart rate dynamics including time-domain, frequency-domain and non-linear domain modifications.

We analyzed the time-domain parameters of HRV before diet administration, at week 14 and at week 28. We found a decrease in the SDHR at week 28 in the MetS group, which is in agreement with other clinical studies that used short-time recordings in patients diagnosed with MetS [15,16,17], without apparent modifications in other standard time-domain parameters. Previous studies found that SDNN was consistently reduced when one or more risk factors were present as compared to none or smaller number of MetS components, however, the best discrimination of MetS was provided by non-linear complexity and irregularity analyses of the RR series in agreement with our results [4,18,19].

Regarding frequency-domain analysis, several changes were found in the lower frequency bands in the MetS group: a decrease in LnVLF at week 28 and an increase in LnLF at both week 14 and week 28. Regarding the former, the results in our experimental model are comparable to those found in the clinical setting where a decrease of VLF was found in humans [16,20,21]. VLF band is linked to sympatho-vagal activity, sympathetic activity reduces the VLF band component while vagal activity increases it [22]. Even though there is uncertainty about the mechanisms responsible for activity within this band, this component is directly correlated with SDHR and it has been related to the heart’s intrinsic nervous system activity [13]. VLF has been used as a predictor of prognosis, linked to cardiovascular disease and MetS; however, the mechanisms of those associations are not well understood [23]. Furthermore, low VLF power has been associated with arrhythmic death more than all-cause death, a correlation that was stronger than LH and HF components [24], which we hypothesise could point towards a possible mechanism for the increased incidence of SCD in MetS, athough we did not explore this evidence in detail in our experiments.

We observed a significant increase in LnLF components already from week 14. With respect to LF, most of the studies have reported a decrease [14,16,20,25] or no changes in this frequency band [17]. Since, in resting conditions, LF power reflects baroreflex activity and not sympathetic activity [26], the increased LF power that we found could be related to alterations in baroreflex control. Results about HF and LF/HF are more heterogeneous. We found no changes in HF, in line with several studies performed in patients [20,25], whereas others reported a decrease of this parameter [14,16,17]. On the other hand, LF/HF balance was also unaltered in our experiments in MetS animals, similar to Koskinen et al. [14] and Stuckey et al. [25], but other studies have observed either increased [17] or decreased [20] LF/HF. Strong discrepancies between studies regarding frequency-domain parameters might be due to heterogeneity in ECG duration, which has a great impact in non-parametric spectral methods, and further, that not all the studies distinguished between VLF and LF bands.

Finally, we studied the non-linear dynamics of the RR time series. Although there is still some uncertainty about the physiological significance of nonlinear parameters for HRV analysis, these measures have shown to be useful for the assessment of cardiovascular risk and sudden cardiac death [5] and are a well-recognized way to distinguish between physiological and pathological conditions [18]. We analyzed entropy-derived physiological markers and our results showed that MetS animals presented an increase of ApEn, SampEn, MSE_min_, MSE_max_ and CI_1–20_. In agreement with previous studies [4], we observed that entropy measures, as well as the complexity index derived from MSE analyses had a great impact of discrimination among the groups at weeks 14 and 28. Both ApEn and SampEn quantified the irregularity and randomness of a time series, the latter being less biased and more reliable when obtained with short length time series [5]. MSE, on the other hand, is a measure of the complexity of physiological time series, taking into account the complex temporal fluctuations conveyed in different scales. Derived from MSE, the CI_1–20_, which includes the global entropy of all 20 scales, can be computed [18]. Unfortunately, studies that have analyzed the nonlinear dynamics and the complexity of HRV using short-term RR time series in MetS are scarce. Nevertheless, collectively, the increase in the different measures of entropy found in our study indicates low predictability of cardiac fluctuations and higher randomness and complexity in cardiac activity, which other researchers have related to pathological states such as myocardial infarction, arrhythmias, sinus sick syndrome, or mutations in intracellular Ca^2+^ or surface membrane ion channel proteins [27,28]. 

The main limitation of our study is the use of short-term RR interval time series, since cardiac autonomic nervous system function was only partially captured. Yet, altered sympathovagal balance can be inferred by both short-term and long-term HRV given its predictive power [5,18]. In order to explore all the changes that MetS produces in cardiac activity, future studies should aim to study long-term time series (i.e., 24 h), which would be necessary to identify all aspects of abnormal autonomic regulation that could be produced by MetS. Furthermore, information on the short-term complexity of HRV analysis in MetS is almost non-existent. Most of the studies have been performed using holter recordings with long-term analyses. This is of crucial importance for interpretation since there are differences in the same parameters between short-term and 24-h HRV in the same population of patients [19]. On the other hand, the main strengths of the study are (1) the use of a diet-induced model in rabbits, which exhibited many of the characteristics of the pathology in humans [8], (2) the controlled experimental conditions and working with experimental models is useful to avoid the interference of confounding factors in a more controlled environment, which are more present in clinical studies and could account for the heterogeneous results found in patients, and (3) we only used male NZW rabbits, but future studies should take gender into account as a factor, given that some changes have been described between men and women [19].

## 5. Conclusions

The aim of this study was to investigate the changes produced in short-term HRV by means of the study of RR autonomic oscillations during the course of MetS induction using high-fat and high-sucrose diets in NZW rabbits. Our results showed that (1) in the time-domain, SDHR was reduced in MetS animals, (2) in the frequency domain, MetS animals presented a decreased VLF, which has been associated with arrhythmic death, and an increased LF, which could point towards alterations in baroreflex activity, (3) the complexity and irregularity of cardiac activity increased in animals with MetS, as depicted by the higher ApEn, SampEn, MSI_min_, MSI_max_ and CI_1–20_, (4) since short-term HRV analysis was used, cardiac autonomic nervous system function was only partially captured and long-term analysis would be necessary to identify all aspects of abnormal autonomic regulation that could be produced by MetS.

## Figures and Tables

**Table 1:** Time-domain parameters of short-term heart rate variability (HRV) analysis.

Parameter	Pre-Test		14 Weeks		28 Weeks	
	Control	MetS	Control	MetS	Control	MetS
NN (ms)	229 (26)	237 (28)	226 (35)	242 (42)	226 (17)	254 (24)
SDNN (ms)	10 (7.2)	10 (5.9)	9 (6.5)	8 (4.8)	12 (8.3)	8 (3.7)
HR (beats/min^−1^)	265 (25)	257 (32)	271 (37)	254 (42)	268 (19)	238 (24)
SDHR (beats/min^−1^)	10.8 (5.2)	11.3 (6)	9.8 (3)	8.4 (4)	13.2 (7)	6.9 (2.6) *^,#^
RMSSD (ms)	4.3 (3.3)	7.4 (5.8)	3.8 (3.2)	4.1 (2.8)	6.6 (7.9)	4.1 (2.9)
NN50 (count)	1.9 (1.6)	18 (48.8)	1 (1.5)	0.5 (0.8)	43.6 (125)	2.8 (6.8)
Triangular index (ms)	2.5 (0.5)	2.9 (0.9)	2.4 (0.8)	2.5 (0.8)	3.1 (1)	2.4 (0.6)
TINN (ms)	59 (17)	73 (47)	65 (40)	45 (20)	59 (37)	55 (39)

Control *n* = 9, MetS *n* = 13. * *p* < 0.05 vs. control. ^#^ *p* < 0.05 vs. pre-diet.

**Table 2:** Frequency-domain parameters of short-term HRV analysis.

Parameter	Pre-Diet		Week 14		Week 28	
	Control	MetS	Control	MetS	Control	MetS
LF (n.u.)	66.0 (26.4)	56.3 (20.1)	56.0 (18.8)	64.8 (13.1)	60.4 (20.1)	68.3 (6.7)
HF (n.u.)	30.8 (19.2)	43.4 (20.4)	43.4 (18.5)	35.1 (13.1)	38.9 (19.8)	31.4 (6.6)
LF/HF (a.u.)	2.4 (1.6)	1.8 (1.3)	1.1 (0.4)	2.3 (1.4)	2.4 (1.6)	2.3 (0.6)
Ln total power (ms^2^)	3.7 (1.3)	3.2 (1.6)	3.6 (1.3)	3.2 (1.2)	3.6 (1.8)	3.3 (1.1)

Ln = natural logarithm. Control *n* = 9, MetS *n* = 13.

**Table 3:** Nonlinear parameters of short-term HRV analysis.

Parameter	Pre-Diet		Week 14		Week 28	
	Control	MetS	Control	MetS	Control	MetS
SD1(ms)	3.1 (2.3)	5.2 (4.1)	2.7 (2.3)	2.9 (2)	4.7 (5.6)	2.9 (2)
SD2 (ms)	13.8 (10.5)	12.4 (8.5)	12.5 (9.4)	11.3 (6.9)	15.8 (11.2)	10.5 (5)
DFA-α1 (a.u.)	0.8 (0.6)	0.6 (0.3)	0.9 (0.6)	0.8 (0.2)	0.8 (0.4)	1 (0.2)
DFA-α2 (a.u.)	0.8 (0.5)	0.7 (0.4)	0.8 (0.5)	0.8 (0.3)	0.7 (0.4)	0.9 (0.2)
ShanEn (a.u.)	3.3 (1)	3.2 (0.6)	3.7 (1)	3.5 (0.4)	3.7 (0.8)	3.7 (0.5)
DET (%)	98.2 (1.9)	97.6 (1.2)	98.1 (1.6)	98.2 (1.3)	98 (2.2)	98.1 (3)
REC (%)	39.3 (13.6)	31.9 (10.8)	38.7 (11.2)	33.9 (11.9)	36.2 (13.9)	38.2 (12.8)

Control *n* = 9, MetS *n* = 13.

## Appendix A

**Table A1:** Description of time-domain HRV measurements.

Parameter	Description
NN (ms)	Mean of the selected R-R interval series.
SDNN (ms)	Standard deviation of the R-R interval series (overall variability).
HR (beats·min^−1^)	Mean heart rate.
SDHR (beats·min^−1^)	Standard deviation of spontaneous heart rate values.
RMSSD (ms)	The root mean square of differences of successive R-R intervals.
NN50 (count)	Number successive R-R interval pairs that differences between successive R-R intervals.
Triangular index (ms)	Integral of the sample density distribution of R-R intervals divided by the maximum of the density distribution.
TINN (ms)	Triangular interpolation of the R-R interval histogram. Baseline width of the minimum square difference triangular interpolation of the maximum of the sample density distribution of R-R intervals.

**Table A2:** Description of frequency-domain HRV measurements.

Parameter	Description
VLF, LF, HF power (%)	Relative powers in VLF, LF and HF ranges. ▪ VLF = VLF/Total power × 100. ▪ LF = LF/Total power × 100. ▪ HF = HF/Total power × 100.
Total power	Total spectral power
LF/HF	Ratio between powers in LF and HF bands.

**Table A3:** Description of non-linear HRV measurements.

Parameter	Description
SD1 (ms)	Standard deviation of the perpendicular point along the line of identity of the Poincaré plot. It represents the instantaneous beat-to-beat short-term variability.
SD2 (ms)	Standard deviation of the perpendicular point along the line of identity of the Poincaré plot. It represents the instantaneous beat-to-beat long-term variability.
DFA α1 (a.u.)	Short terms fluctuations (4–12 beats) of detrended fluctuation analysis. The slopes of a log-log plot (correlation measure as a function of segment length).
DFA α2 (a.u.)	Long-terms fluctuations (13–64 beats) of detrended fluctuation analysis. The slopes of a log-log plot (correlation measure as a function of segment length).
ApEn (a.u.)	Approximate entropy, measures the complexity of RR time series (*m* = 2, *r* = 0.2) [29].
SampEn (a.u.)	Sample entropy, measures the irregularity RR time series (*m* = 2, *r* = 0.2) [30].
MSEmin (a.u.)	Minimum value of multiscale entropy [31].
MSEmax (a.u.)	Maximum value of multiscale entropy [31].
CI_1–20_ (a.u.)	Mean of entropies on all 20 scales of MSE [25].
DET (%)	Determinism (percentage of recurrence points which form diagonal lines in recurrence plot)
REC (%)	Recurrence rate (percentage of recurrence points in recurrence plot)
